# A Multimodal Neurophysiological Approach to Evaluate Educational Contents in Terms of Cognitive Processes and Engagement

**DOI:** 10.3390/bioengineering12060597

**Published:** 2025-05-31

**Authors:** Vincenzo Ronca, Pietro Aricò, Luca Tamborra, Antonia Biagi, Gianluca Di Flumeri

**Affiliations:** 1 Department of Computer, Control, and Management Engineering, Sapienza University of Rome, 00185 Rome, Italy; pietro.arico@uniroma1.it; 2 Department of Anatomical, Histological, Forensic and Orthopaedic Sciences, Sapienza University of Rome, 00185 Rome, Italy; luca.tamborra@uniroma1.it; 3 Department of Technological Innovations and Safety of Plants, Products and Anthropic Settlements, Istituto Nazionale per L’Assicurazione Contro Gli Infortuni Sul Lavoro (INAIL), 00144 Rome, Italy; a.biagi@inail.it; 4 Department of Molecular Medicine, Sapienza University of Rome, 00185 Rome, Italy; gianluca.diflumeri@uniroma1.it

**Keywords:** neurophysiology, learning, education, mental states, EEG

## Abstract

Background: Understanding the impact of different learning materials in terms of comprehension and engagement is essential for optimizing educational strategies. While digital learning tools are increasingly used, offering and multiplying different educational solutions, their effects on learners’ mental workload, attention, and engagement remain underexplored. This study aims to investigate how different types of learning content—educational videos, academic videos, and text reading—affect cognitive processing and engagement. Methods: Neurophysiological signals, including electroencephalography (EEG), electrodermal activity (EDA), and photoplethysmography (PPG), were recorded from experimental participants while they were engaged with each learning content. Subjective assessments of cognitive effort and engagement, together with a quiz to assess the knowledge acquisition, were collected through questionnaires for each tested content. Key neurophysiological metrics, such as engagement and Human Distraction Index (HDI), were computed and compared across conditions. Results: Our findings indicate that video-based learning materials, particularly educational videos with visual enhancements, elicited higher engagement and lower cognitive load compared to text-based learning. The text reading condition was associated with increased mental workload and a higher distraction index, suggesting greater cognitive demands. Correlation analyses confirmed strong associations between neurophysiological indicators and subjective evaluations. Conclusions: The results highlight the potential of neurophysiological measures to objectively assess learning experiences, paving the way for designing more effective and engaging learning platforms.

## 1. Introduction

The quest to understand and optimize the learning process is a cornerstone of educational research. Unraveling the cognitive mechanisms underlying knowledge acquisition not only enhances theoretical understanding but also paves the way for more effective, personalized, and inclusive teaching methodologies. By integrating insights from neuroscience, psychology, and educational technology, researchers can develop personalized learning strategies that cater to diverse learner needs, ultimately improving educational outcomes. While traditional assessment methods like tests and questionnaires provide valuable information, they offer a limited window into the dynamic, internal cognitive, and affective states that shape learning. To gain a deeper understanding, researchers are increasingly turning to neurophysiological measures, capturing the subtle physiological changes that accompany relevant cognitive processes of the learner, such as mental effort, attention, engagement, and distraction. This approach, often termed educational neuroscience, promises to provide objective, real-time insights into the learner’s experience, moving beyond subjective self-reports and behavioral observations [1,2].

The application of neurophysiological signals-based approaches, such as the ones based on electroencephalography (EEG), electrodermal activity (EDA), and photoplethysmography (PPG), in real-world educational settings is gaining constant interest among the scientific community [3,4]. Unlike fMRI or other neuroimaging techniques that require constrained laboratory environments, EEG, EDA, and PPG are relatively portable and less sensitive to movement artifacts, making them suitable for studying learning in more naturalistic contexts. This is crucial for capturing the complexities of real-world learning experiences, which often involve dynamic interactions and a variety of stimuli. Researchers have begun to use these tools to investigate learning in classrooms, online environments, and even during outdoor activities [5,6,7,8]. The existing literature demonstrates the potential of these neurophysiological signals to reveal subtle differences in cognitive and affective states during various learning tasks. Studies have shown that EEG can differentiate between levels of mental workload during problem-solving [9,10,11], identify periods of inattention during lectures [12,13,14], and even predict learning outcomes based on neural activity patterns [15,16,17]. EDA and PPG have been used to assess stress levels during exams, monitor engagement during interactive simulations [15,18], and detect emotional responses to different types of learning materials [16,19,20,21].

However, a comprehensive investigation of how different types of learning materials, commonly used in real-world educational settings, impact cognitive responses from a neurophysiological point of view remains an area to be explored [22,23,24,25]. Specifically, the comparative effects of visually rich educational videos, interactive training videos designed to promote skill acquisition, and traditional text-based learning materials on students’ cognitive and affective states need to be systematically examined using a unified neurophysiological framework. Many existing studies focus on a single modality (e.g., only EEG) or compare very dissimilar learning activities, making it difficult to draw direct comparisons. Additionally, these previous approaches rely on neurophysiological signal collection equipment that is not fully compatible with real-life contexts. This study aims to address these gaps by investigating the neurophysiological impact of three common learning contents: an educational learning video with advanced graphic solutions and a simpler communicative language style, a more traditional academic content based on a PowerPoint presentation with a voice-over, and classical text reading. The overarching objective is to objectively characterize the distinct neurophysiological reactions associated with each learning content, using a multimodal neurophysiological approach and, more importantly, to provide objective insights about which one could be the most engaging and efficient learning environment, through a multimodal neurophysiological approach compatible with real-life contexts. This investigation was designed to be performed by neurophysiologically characterizing distraction and engagement, two of the most crucial aspects in learning and education. More specifically, the present research assessed the reliability of a Human Distraction Index (HDI), previously validated in a different context [26], and an Engagement index [5,27,28]. By simultaneously recording brain activity, electrodermal and cardiovascular responses, it was aimed to capture the interplay between cognitive workload, attentional focus, and engagement elicited by each type of material.

## 2. Materials and Methods

### 2.1. Participants

Ten volunteers from Sapienza University, including Master thesis students, Ph.D. candidates, and staff members, ranging in age from 24 to 37 years (M = 28.6, SD = 4.56) and with a similar and strong technical background, were involved in the presented study. They participated in the study without any reward. The experimental task consisted of using the three selected educational contents, related to the same topic, maintaining focus and striving to absorb as much information as possible, as they were informed of a subsequent questionnaire. Before starting, each participant received a clear explanation of the study’s procedures and provided written informed consent. While the overall procedure was explained, the specific focus on comparing cognitive engagement across different material types was not explicitly highlighted to avoid influencing their natural viewing behavior. Following the observation period, a comprehensive debriefing session clarified the study’s full objectives. Permission to use any visual recordings from the session was also secured. This research was conducted in accordance with the Declaration of Helsinki (1975, revised in 2008) and received ethical approval from the Sapienza University of Rome ethics committee (protocol code 2024/03-002 approved on 21 March 2024).

### 2.2. Experimental Protocol

This study presented each participant with three distinct educational contents concerning a single topic: Bluetooth wireless technology. This topic was chosen for its broad relevance in order to avoid any bias due to individual interests. Limiting the study to a single topic was deemed appropriate for this initial investigation. The three educational contents were as follows:

- Task “*Educational video*”: A popular science video (6 min 49 s) incorporating short video clips, infographics, and practical examples (https://www.youtube.com/watch?v=3MUstQxdi34, accessed on 5 May 2024).
- Task “*Academic video*”: A traditional lecture-style video (7 min 17 s) featuring PowerPoint slides and a voice-over narration (https://www.youtube.com/watch?app=desktop&v=eV4ufhg7W6c, accessed on 5 May 2024).
- Task “*Text reading*”: An encyclopedic text excerpt, designed to be read within approximately 7 min (https://it.wikipedia.org/wiki/Bluetooth, accessed on 5 May 2024).

**Figure 1:**
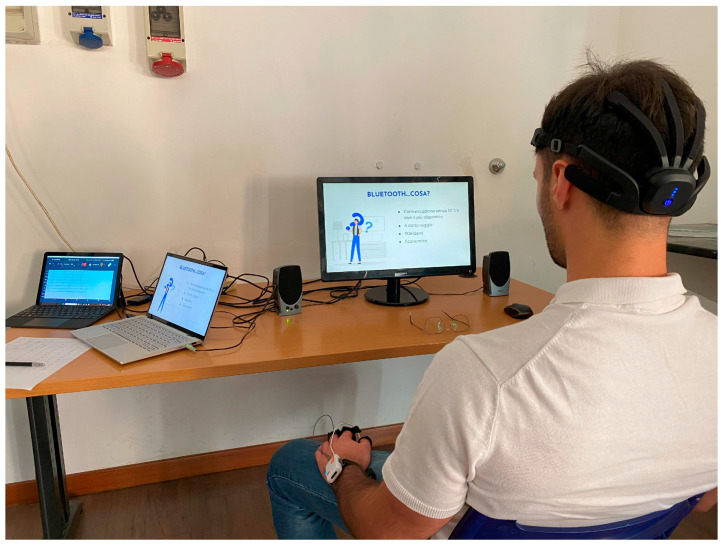
Experimental settings. The participant sat in front of the PC to provide learning materials and wore the EEG, PPG, and EDA signals collection equipment.

Participants performed each task individually, seated comfortably in front of a computer monitor with audio delivered via external speakers, inside a standard-like classroom in order to simulate an educational environment (Figure 1). The order of presentation of the three contents was randomized across participants to mitigate potential order effects, such as habituation or expectation. Neurophysiological data were recorded continuously from each participant during each task. Prior to the start of each experimental session, a 60 s resting-state baseline was recorded for each participant at their workstation. A five-minute break between each learning material access was foreseen.

### 2.3. Neurophysiological Data Collection and Processing

#### 2.3.1. Electroencephalography (EEG)

During the experimental tasks, the participants’ cerebral activity (EEG) was captured using the Mindtooth Touch EEG wearable system (Brain Products GmbH, Gilching, Germany & BrainSigns srl, Rome, Italy, [29]). The device embeds eight recording EEG channels, placed over the prefrontal and parietal regions, specifically at the AFz, AF3, AF4, AF7, AF8, Pz, P3, and P4 locations, in accordance with the 10-10 International System, reference and ground specifically on the right and left mastoids, and sample rate of 125 Hz. All the electrodes’ impedances were kept below 100 (kΩ), and the quality of the EEG signals was checked before and during the experimental protocol, while the electrodes’ positioning was initially confirmed through scalp distance measurements.

Initially, a pre-processing phase was carried out to identify and correct both physiological and non-physiological artifacts unrelated to the cerebral activity of interest, such as ocular, muscular, and movement-induced signals. In this regard, the EEG signal was band-pass filtered with a 5th-order Butterworth filter in the interval 2–30 (Hz), beside a 50 Hz-notch filtering. The eye blink artifacts were detected and corrected by employing the o-CLEAN method [30], which corresponds to a novel method combining regression and multi-channel adaptive filtering for accurately identifying and correcting ocular-based artifacts. For further sources of artifacts, such as the ones derived from muscular activity and movements, ad hoc algorithms based on the EEGLAB toolbox [31] were applied. More specifically, one statistical criterion was applied to the 1 s-long pre-processed EEG signal. Firstly, EEG epochs with the signal amplitude exceeding ±80 μV were marked as “artifacts’’. Secondly, such EEG epochs marked as “artifacts” were removed from the EEG dataset in order to proceed with the EEG processing by considering exclusively the cleaned channels. All the EEG channels were preserved with an average artifact percentage below 8%, as shown in Table 1.

Once the EEG pre-processing steps were completed, the Global Field Power (GFP) was calculated for the EEG frequency band of interest for computing the mental states on which the present study focused. Therefore, the EEG GFP features were computed within the Theta, Alpha, and Beta frequency bands. It must be underlined that the GFP was chosen as the parameter of interest describing brain EEG activity since it has the advantage of representing, within the time domain, the degree of synchronization on a specific cortical region of interest in a specific frequency band [32,33]. In terms of technical implementation, the GFP was mathematically computed according to the approach described by Vecchiato and colleagues [34]. Concerning the EEG GFP features computation, the frequency bands were defined according to the Individual Alpha Frequency (IAF) value [35] computed for each participant. In order to compute the IAF, a 60 s-long experimental condition was collected while the participants kept their eyes closed, since the Alpha peak is consistently prominent in such a condition. Subsequently, the EEG GFP was computed over all the EEG channels for each 1 s-long epoch through a Hanning window of the same length (1 s, which means 1 Hz of frequency resolution according to the time resolution required from the presented approach) as follows:

$${GFP}_{band,\;region}=\frac{1}{N}\;\sum_{i=1}^{N}x_{i,\;band}^{2}(t),$$

where *N* is the number of the considered EEG channels and $x_{i,\;band}^{2}$ is the *i*-th EEG channel filtered within the selected EEG frequency band. After the EEG data preprocessing, the EEG *GFP*-derived features were computed according to the research objectives. More specifically, the mental workload, attention, and cognitive engagement were computed according to the following:

$$Mental\;workload=\frac{Frontal\;Theta_{GFP}}{Parietal\;Alpha_{GFP}}=\frac{\frac{1}{5}\;\sum_{i=1}^{5}x_{i,\;theta}^{2}(t)}{\frac{1}{3}\;\sum_{i=1}^{3}x_{i,\;alpha}^{2}(t)},$$

$$Attention=\frac{Frontal\;Beta_{GFP}}{Frontal\;Theta_{GFP}}=\frac{\frac{1}{5}\;\sum_{i=1}^{5}x_{i,\;beta}^{2}(t)}{\frac{1}{5}\;\sum_{i=1}^{5}x_{i,\;theta}^{2}(t)},$$

$$Engagement=\frac{Parietal\;Beta_{GFP}}{Parietal\;Theta_{GFP}+Parietal\;Alpha_{GFP}}=\frac{\frac{1}{3}\;\sum_{i=1}^{3}x_{i,\;beta}^{2}(t)}{\frac{1}{3}\;\sum_{i=1}^{3}x_{i,\;theta}^{2}\left(t\right)+\frac{1}{\;3}\;\sum_{i=1}^{3}x_{i,\;alpha}^{2}(t)},$$

where $Frontal\;Theta_{GFP}$ and $Frontal\;Beta_{GFP}$ were computed by considering the AFz, AF3, AF4, AF7, and AF8 EEG channels, while the $Parietal\;Theta_{GFP}$, $Parietal\;Alpha_{GFP},Parietal\;Beta_{GFP}$ were computed by considering the Pz, P3, and P4 EEG channels. In this context, the mental workload index calculation was based on prior research using EEG to assess mental workload [7,36,37,38]. The Attention index was defined as the inverse of the Theta–Beta Ratio, an established EEG marker of attentional deficits in Attention Deficit Hyperactivity Disorder (ADHD) [39,40] and a validated measure of distributed attention during concurrent task performance [41,42]. The Engagement index was defined according to prior scientific works, which validated the cognitive aspect of engagement in a similar learning context [28]. Subsequently, the above-described neurophysiological metrics were z-score normalized according to the eyes open condition, considered as baseline. Then, the mental workload and attention metrics were furtherly combined to compute the Human Distraction index (HDI), based on the assumption that a high workload does not necessarily imply task engagement, since mental resources can also be devoted to activities not related to the task being performed (i.e., mind wandering [43,44,45]. Such an index was already successfully proposed by Ronca and colleagues [26] in a highly realistic simulated driving context. Such an index was defined according to the following:

$$HDI=Mental\;workload-Attention=\frac{Frontal\;Theta_{GFP}}{Parietal\;Alpha_{GFP}}-\frac{Frontal\;Beta_{GFP}}{Frontal\;Theta_{GFP}}$$

#### 2.3.2. Photoplethysmography (PPG)

Photoplethysmography (PPG) signals, crucial for evaluating volumetric variations in blood content within biological tissues, were collected using the advanced Shimmer3 GSR + apparatus (Shimmer Sensing, Dublin, Ireland). This instrument was securely positioned on the wrist of the participant’s non-dominant hand, ensuring stable and precise signal capture at a sampling rate of 64 Hz. The PPG datasets underwent a digital filtration process using a 5th-order Butterworth band-pass filter, specifically spanning a frequency range of 1–5 Hz. This filtering protocol was carefully designed to effectively exclude the underlying continuous components and mitigate the influence of any gradual signal drift, whilst accentuating the characteristic pulsatile oscillations inherent in the PPG signal, which are indicative of cardiac activity. To further delve into the detailed cardiac rhythms, the distinguished Pan–Tompkins’s algorithm [46] QRS Detection was deployed. This algorithm is adept at discerning pulse-associated peaks, which subsequently facilitates the computation of the Inter-Beat Intervals (IBI signal). However, the resultant IBI datasets occasionally contained aberrations or artifacts. To address this, the comprehensive HRVAS Matlab (MathWorks Inc., Natick, MA, USA) suite [47] was harnessed to refine and enhance the data quality. Subsequently, these purified IBI signals were algorithmically processed to deduce the Heart Rate (HR), typically represented in the metric “Beats per minute”. To ensure individual specificity and normalization, the derived HR metrics for each participant were systematically adjusted by deducting their personalized baseline HR mean and then normalizing the outcome using their unique HR standard deviation.

#### 2.3.3. Electrodermal Activity (EDA)

Electrodermal activity (EDA) signals, indicative of fluctuations in the skin’s electrical conductance as a consequence of modulations in sweat gland activity, were acquired employing the advanced Shimmer3 GSR+ unit (Shimmer Sensing, Dublin, Ireland), the same cutting-edge apparatus mentioned previously. This device was positioned on the wrist of the participant’s non-dominant hand to guarantee optimal data fidelity, with EDA signals being recorded at a detailed sampling rate of 64 Hz. The raw EDA signals underwent a series of computational refinements. Initially, the signals were passed through a low-pass filter with a discriminative cut-off frequency set at 1 Hz, serving to emphasize the desired frequency components and attenuate unwanted high-frequency noise. Post this filtration step, an advanced artifact correction tool within the Matlab framework (version 2024b) was wisely employed to repair and eliminate any evident signal anomalies, such as sudden discontinuities and unwarranted spikes. To further refine and process these signals, the Ledalab suite [R] was deployed, a specialized open-source software toolbox seamlessly integrated within the Matlab ecosystem (version 2024b), tailored specifically for advanced EDA signal analysis. Employing the principles of continuous decomposition analysis [48], the suite enabled the separation of the EDA signal into its constituent tonic (SCL) and phasic (SCR) components. The SCL, or Skin Conductance Level, represents the gradual and sustained modulations in the EDA signal and predominantly encapsulates the overarching arousal state of the participant. Conversely, the SCR, or Skin Conductance Response, mirrors the transient and rapid fluctuations in the EDA and is conventionally attributed to discrete stimulus-driven physiological reactions. However, given the inherent limitations stemming from the involved devices’ relatively low sampling frequency, this study had to prudently prioritize and focus on the more steady-state SCL component for its analytical rigor. As a final step in the data normalization process, the extracted SCL values for each individual were systematically adjusted to a standard scale. This was achieved by deducting the participant-specific baseline SCL, followed by normalization using the unique SCL standard deviation pertinent to each participant.

### 2.4. Subjective and Behavioral Data Collection

Following each experimental condition, participants completed a five-question quiz designed to assess comprehension and retention of the material presented. Crucially, the quiz questions were specific to the content of each individual modality, preventing potential learning transfer effects across conditions. Practically speaking, even if all three contents were related to the same topic, the quiz was designed in order to be related to information contained only in that specific content. In addition to the comprehension quiz, participants answered five questions evaluating their subjective learning experience, adapted from previously validated questionnaires [49,50]. More specifically, these inquiries solicited participants to assign a rating, on a numerical continuum from 1 to 10, reflecting their subjective experiences concerning:

- The simplicity with which they could comprehend the disseminated information.
- The facility with which they could internalize the content.
- The capacity to sustain attention during the entirety of the task.
- The degree of interest elicited by the employed narrative modality.
- The extent of engagement is provoked by the narrative approach.

In the evaluation process, careful attention was taken to ensure procedural consistency; every participant was presented with an identical set of questions, which bolstered the uniformity and comparability of the assessment metrics.

### 2.5. Statistical Analyses

As a preliminary step, the Shapiro–Wilk test [51] was selected to determine the normality of the distribution related to each of the considered statistical features. In the case of normal distributions, the Analysis of Variance (ANOVA) was selected to compare the two experimental groups (i.e., Expert vs. Novices). If the distributions’ normality was not confirmed by the Shapiro–Wilk test, the Friedman chi-squared test was performed. In case of statistically significant main effect resulting from the group comparisons, the post hoc tests were performed to assess eventual individual differences (i.e., experimental condition vs. another one). For all tests, statistical significance was set at α = 0.05. Additionally, the repeated measures correlation analysis [52] was additionally performed to validate the EEG-based Engagement index with respect to the subjective measurements collected from the participants, both at the single-participant level and in the entire group.

## 3. Results

This section was divided into different subparagraphs in order to organize all the results on the basis of the related data source, i.e., questionnaires (subjective results), learning performance (behavioral results), and neurophysiological ones.

Among all the comparisons, only the analyses providing statistically significant results are reported here.

### 3.1. Neurophysiological Results

The statistical analysis performed on the autonomic-derived parameters, i.e., SCL and SCR computed from the EDA and the PPG-derived features, did not reveal any statistical effect between the tested experimental conditions.

**Figure 2:**
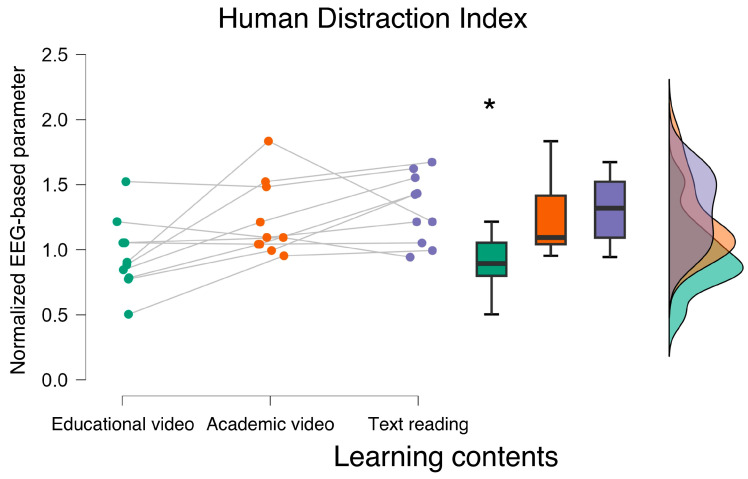
The ANOVA performed on the Human Distraction Index (i.e., HDI) revealed that the neurophysiological distraction evaluation was statistically lower when accessing the educational video material. * indicates the statistical significance (*p* < 0.05).

**Figure 3:**
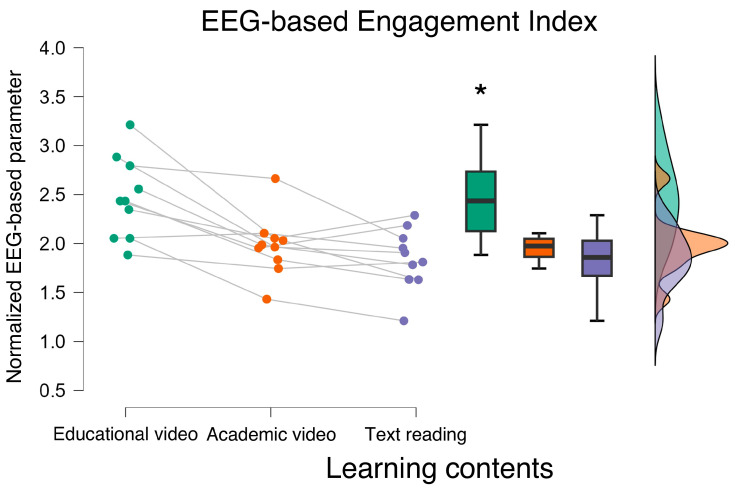
The ANOVA performed on the EEG-based engagement index revealed that participants were neurophysiologically more engaged when exposed to the educational video material. * indicates the statistical significance (*p* < 0.05).

Concerning the EEG-based parameters, ANOVA showed a statistically significant main effect of the experimental conditions in terms of HDI and Engagement index (HDI: F = 6.560, *p* = 0.007; ω^2^ = 0.204; Engagement index: F = 19.641, *p* < 0.001; ω^2^ = 0.357) (Figure 2 and Figure 3). More specifically, the post hoc analysis showed that during the *educational video* condition, participants exhibited the lowest HDI (*p* < 0.01), and the highest Engagement index (*p* < 0.006). No statistically significant differences were observed between the Academic video and text reading conditions in terms of HDI and Engagement index (all *p* > 0.10).

### 3.2. Correlation Analysis

**Figure 4:**
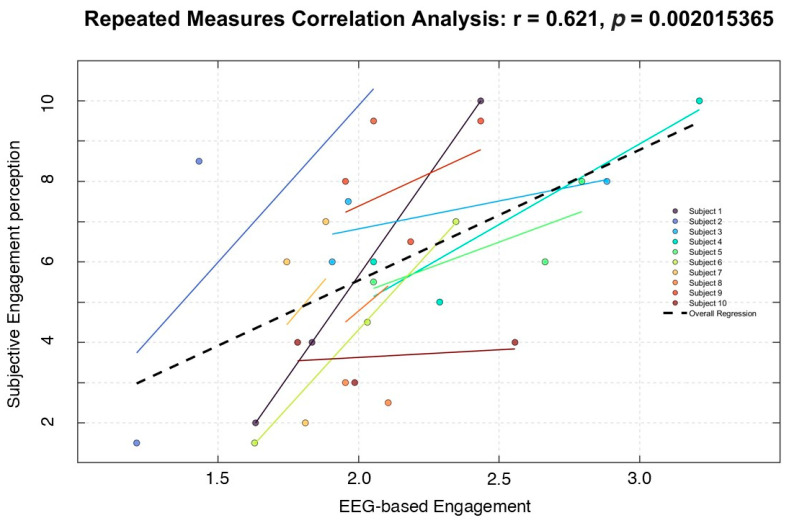
The repeated measure correlation analysis showed that the neurophysiological Engagement index and the respective subjective perceptions exhibited a similar temporal dynamic along the experimental conditions.

Finally, a conclusive statistical analysis was performed to assess the correlation between the neurophysiological measurements related to the impact of the different learning materials and their respective subjective scores provided by the participants across the experimental conditions. More specifically, the repeated measure correlation analysis [52] was performed between the EEG-based Engagement index and its respective subjective score (i.e., EP). As shown in Figure 4, the analysis revealed a strong and significant correlation between these two measurements (R = 0.621; *p* = 0.0002), indicating that the measured cognitive impact of the different learning materials in terms of engagement was temporally coherent with the subjective perceptions of the participants.

### 3.3. Subjective Results

The subjective data collected along the experimental protocol were combined in order to obtain two subjective scores. The first one is associated with the required cognitive resources perception (CRP), computed by averaging the subjective scores related to the first three dimensions of the above-described questionnaire (i.e., the higher the better, since high scores were associated with easy task perceptions). The second one is associated with the subjective engagement perception (EP) (i.e., the higher the better, since high scores were associated with engaging task perceptions), obtained by averaging the latter two dimensions of the above-described questionnaire.

**Figure 5:**
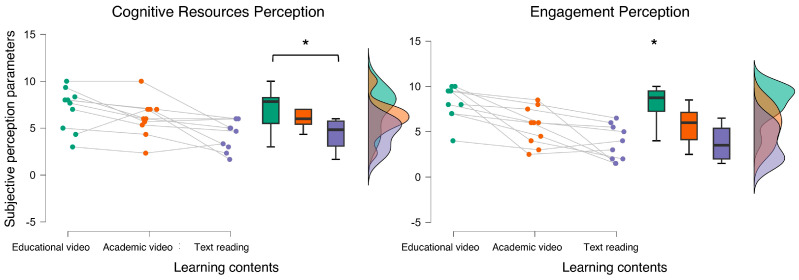
The statistical analysis of the subjective scores revealed that participants perceived as easier and more engaging than the educational video in terms of cognitive impact. * indicates the statistical significance (*p* < 0.05).

The statistical analysis revealed a significant main effect of the experimental condition associated with the subjective CRP and EP scores (CRP: Friedman chi-squared = 10.474, *p* = 0.001, η^2^ = 0.335; EP: Friedman chi-squared = 15.436, *p* < 0.001, η^2^ = 0.459). More specifically, the post hoc analysis showed that the text reading condition was perceived as requiring more in terms of cognitive resources and as less engaging (all *p* < 0.01). Additionally, the post hoc analysis showed that the *educational video* condition resulted in statistically more engaging (*p* < 0.001) compared to the *text reading* one (Figure 5).

### 3.4. Behavioral Results

**Figure 6:**
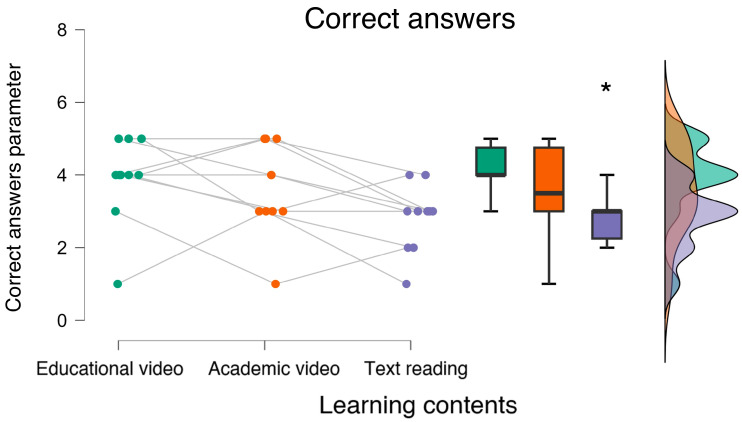
The statistical analysis showed that the participants provided fewer correct answers when accessing the text reading material. * indicates the statistical significance (p < 0.05).

The non-parametric repeated measure analysis performed on the behavioral data, i.e., the correct answers recorded for each experimental condition, revealed that participants committed the highest error rate during the *text reading* condition (Friedman chi-squared = 6.178, *p* = 0.032, η^2^ = 0.116; post hoc: *p* = 0.02) (Figure 6).

## 4. Discussion

This study aimed to neurophysiologically characterize the impact of different learning materials by employing a multimodal approach that integrates EEG, EDA, and PPG recordings, in parallel with subjective measurements. The results demonstrated that different educational contents elicit distinct neurophysiological responses, highlighting how cognitive load, engagement, and attentional mechanisms vary depending on the format of the learning content. More importantly, the present research demonstrates how it is possible to the multimodal neurophysiological modeling of the impact of different learning materials, by proposing an innovative use of biomedical devices—specifically, wearable EEG—in a real-life context.

The findings indicate that video-based learning materials, particularly *educational videos* enriched with visual elements and structured narration, were associated with higher engagement levels, as evidenced by EEG-derived metrics. This is consistent with previous literature suggesting that dynamic and multimodal learning experiences enhance cognitive processing and memory retention. Conversely, the text-based learning condition (i.e., text *reading*) elicited higher cognitive distraction and a lower Engagement index, as reflected in EEG-derived parameters. This aligns with prior research showing that passive reading demands sustained attention and greater cognitive effort, potentially leading to increased mental fatigue [53]. The strong correlation between subjective engagement scores and neurophysiological engagement indices further validates the robustness of the proposed multimodal framework. This consistency between self-reported experiences and objective measures highlights the potential of neurophysiological monitoring as a reliable tool for assessing learning effectiveness in real-world educational settings.

However, some limitations must be acknowledged. The study involved a relatively small sample size, limiting the generalizability of the results. This aspect is particularly relevant when considering the potential discrepancy between the different learning modules. In this regard, an additional statistical analysis was carried out to ensure a similarity between the modules. In particular, the three learning modules were compared by considering their temporal presentation to the participants (i.e., as they were not randomized). The statistical analysis revealed an increase in the participants’ performance in terms of correct answers, resulting in a marginal statistical increase (*p* = 0.07) in the correct answers from accessing the first module to accessing the third one. Indeed, future research must include a larger sample size in order to independently assess subject matter retention and, possibly, assess the reliability of the proposed approach by considering different age ranges and sample sizes. Additionally, the experimental design focused on a single topic, which may not fully capture the variability of different subject matters. Future research should expand the dataset, include a broader range of learning materials and populations characterized by a wider scale range, since the scientific literature indicates that age-related differences in terms of EEG patterns could occur [54], and explore individual differences in cognitive responses. In this regard, it has to be underlined that the proposed HDI and Engagement indices would benefit from a wider and transversal validation. Finally, another interesting aspect to be considered through the presented neurophysiological approach consists of assessing the influence of group interaction on learning processes [28].

Despite these limitations, this study represents a step forward in understanding how learning materials shape cognitive and engagement, paving the way for more personalized and effective digital learning experiences.

## 5. Conclusions

This study investigated the impact of different learning materials on specific cognitive processes by employing a multimodal approach that integrated EEG, EDA, and PPG recordings with subjective measurements. The findings demonstrated that different educational modalities elicit distinct neurophysiological responses, providing objective insights into how cognitive load, attentional mechanisms, and engagement vary depending on the format of the learning content. This opens the way for the future development of adaptive technological solutions based on Brain–Computer Interface principles. The neurophysiological modeling of the impact of different learning materials consisted of a first investigation and demonstration of how an EEG-based approach is reliable in objectively assessing the brain mechanisms modulations in education. Given its exploratory nature, the work focused on a simple environment, but the observed results pave the way to further integrate such an approach in more complex learning scenarios.

The results underscore the potential of neurophysiological measures to optimize learning experiences by providing real-time, objective assessments of cognitive processes specifically related to learning. More importantly, the proposed multimodal approach relies on wearable equipment for collecting neurophysiological signals, coupled with signal processing methods fully compatible with real-world applications [6,55,56,57,58].

### Future Trends

The effectiveness of the proposed neurophysiological framework in distinguishing between different learning conditions highlights its potential applicability in adaptive learning environments. By integrating real-time neurophysiological feedback, future e-learning systems could dynamically adjust content delivery to optimize learning efficiency and reduce cognitive overload.

Future e-learning platforms could leverage this approach to dynamically adjust instructional materials based on individual cognitive responses, enhancing learning efficiency and reducing distractive behaviors [59,60,61,62]. In conclusion, this research represents a preliminary, but significant step forward in understanding how different learning materials impact cognitive and engagement, paving the way for more adaptive and personalized educational experiences.

## Figures and Tables

**Table 1:** Mean artifact percentage for each participant over all the EEG channels.

Subject	Mean Artifact (%)
Participant 1	5.88
Participant 2	6.93
Participant 3	7.41
Participant 4	6.89
Participant 5	7.71
Participant 6	6.22
Participant 7	7.09
Participant 8	6.57
Participant 9	7.81
Participant 10	6.02

## Data Availability

The data that support the findings of this study are available from the corresponding authors upon reasonable request. The data are not publicly available since they are biometric data and they are considered sensitive data as of EU GDPR n. 2016/679.
